# Reactive Fabrication and Effect of NbC on Microstructure and Tribological Properties of CrS Co-Based Self-Lubricating Coatings by Laser Cladding

**DOI:** 10.3390/ma11010044

**Published:** 2017-12-28

**Authors:** Liuyang Fang, Hua Yan, Yansong Yao, Peilei Zhang, Qiushi Gao, Yang Qin

**Affiliations:** 1 School of Materials Engineering, Shanghai University of Engineering Science, Shanghai 201620, China; fangliuyangs@163.com (L.F.); 18800237069@163.com (Y.Y.); peilei@sues.edu.cn (P.Z.); 13141526618@163.com (Q.G.); qinyang0305@163.com (Y.Q.); 2 Shanghai Collaborative Innovation Center of Laser Advanced Manufacturing Technology, Shanghai University of Engineering Science, Shanghai 201620, China

**Keywords:** laser cladding, metal matrix self-lubricating coating, microstructure, tribological behavior

## Abstract

The CrS/NbC Co-based self-lubricating composite coatings were successfully fabricated on Cr12MoV steel surface by laser clad Stellite 6, WS_2_, and NbC mixed powders. The phase composition, microstructure, and tribological properties of the coatings ware investigated by means of X-ray diffraction (XRD), scanning electron microscopy (SEM), and energy dispersive spectrometer (EDS), as well as dry sliding wear testing. Based on the experimental results, it was found reactions between WS_2_ and Co-based alloy powder had occurred, which generated solid-lubricant phase CrS, and NbC play a key role in improving CrS nuclear and refining microstructure of Co-based composite coating during laser cladding processing. The coatings were mainly composed of γ-Co, CrS, NbC, Cr_23_C_6_, and CoC_x_. Due to the distribution of the relatively hard phase of NbC and the solid lubricating phase CrS, the coatings had better wear resistance. Moreover, the suitable balance of CrS and NbC was favorable for further decreasing the friction and improving the stability of the contact surfaces between the WC ball and the coatings. The microhardness, friction coefficient, and wear rate of the coating 4 (Clad powders composed of 60 wt % Stellite 6, 30 wt % NbC and 10 wt % WS_2_) were 587.3 HV_0.5_, 0.426, and 5.61 × 10^−5^ mm^3^/N·m, respectively.

## 1. Introduction

The lubricating and tribological property of moving components under extreme conditions such as high temperature, heavy load, vacuum, and humid and dry air environments seriously affect their reliability and service time of the entire system [1,2]. However, traditional oils or other liquid lubricants easy fail in these severe conditions [3]. The metal matrix self-lubricating composites are highly desirable for their remarkable properties [1,4]. Those materials possess a low friction coefficient and good wear resistance under the condition of lubricant failure or no lubrication media, and have been successfully used in aerospace systems, steam turbines, and industrial drying furnaces [1,4,5,6,7]. In general, metal matrix self-lubricating materials are utilized in a form of coating instead of bulk composite, since the friction/wear always takes place on work-piece surfaces [8], and various surface modification techniques can be employed to produce metal matrix self-lubricating coatings (MMSCs), such as plasma spraying [9,10], electroplating [11,12], physical vapor deposition [13], and laser cladding [14,15], among which laser cladding is very effective and offers distinct advantages such as lower porosity, a narrow heat-affected zone, and strong metallurgical bonding.

As a well-known solid lubricant, WS_2_ has a lamellar structure much like MoS_2_ and graphite, is easily used to form transfer lubricious films between the friction pair interface, and is used as a solid lubricant additive. However, previous research [14,16,17] has shown that WS_2_ decomposed and oxidized when it was used as solid lubricant additives to fabricate MMSCs by laser cladding. In order to overcome the difficulties, many attempts have been tried to inhibit its decomposition. Wang et al. [16] reported the high-energy ball milling method to encapsulate nano-Ni onto submicron WS_2_ to prevent the mass loss of WS_2_ during laser cladding. Liu et al. [17] applied the electroplating technique that encapsulated WS_2_ with a layer of micro Ni-P to inhibit its decomposition during the laser cladding process. Although the encapsulating methods and optimization of the laser cladding process parameters could have decreased the decomposition of WS_2_ to some extent, there was still a lot of WS_2_ decomposed and vaporized during the laser cladding processing. These problems weaken the performance of laser clad self-lubricating coating. 

Recently, the solid-lubricant phase was synthesized using the high-energy method that shows promise for producing MMSCs [8,18,19,20], which could avoid the decomposition and oxidization of lubricants [8]. Skarvelis et al. [18] fabricated a MMSC containing TiS and Ti4C_2_S_2_ on AISI 1522H plain steel substrate by plasma transferred arc of TiS_2_ and TiC powder mixture. Results showed that the formation of TiS and Ti4C_2_S_2_ in the coating was beneficial to the coating’s tribological properties. Lu et al. [19] fabricated the Ti_2_CS/CrS MMSC by laser clad NiCr/Cr_3_C_2_-WS_2_ mixed powders; the results indicated that the composite coating presented excellent self-lubricating properties due to the formation of the lubricious CrS and Ti_2_CS sulfides. Among those studies, the friction-reducing role of self-lubrication phases (such as TiS, Ti_2_CS, and CrS) has been investigated in detail and the formation mechanism of CrS is also involved. It is worth mentioning that the above researches do not consider the effect of ceramic particles on the formation mechanism of CrS. Moreover, the comprehensive effect of CrS and ceramic particles on the wear mechanism also needs to be revealed.

In the present study, the objective is focusing on the reaction synthesis that fabricates the self-lubricating composite coating that contains solid-lubricant phase CrS, as well as exploring the idea that ceramic particles contribute to improving the microstructural evolution of the coating. Besides, the tribological behavior of the coatings was also explored. Here, a novel method was developed to fabricate the coating that contains CrS and ceramic particles. Co-based alloy powder (Stellite 6) was introduced into laser cladding, together with WS_2_ and NbC. WS_2_ was selected to synthesize CrS with Stellite 6, while the ceramic particle of NbC was selected to reinforce the coating and improve CrS formation. For comparison, the Stellite 6, WS_2_/Stellite 6, and NbC/Stellite 6 coatings were also prepared by laser cladding. The microstructure and wear performance of those coatings are presented and discussed.

## 2. Materials and Methods

### 2.1. Materials

Flat plate of unheated-treated Cr12MoV steel with the dimension of 50 mm × 50 mm × 10 mm was selected as substrate. According to Xiao-Long Lu’s [19] research, WS_2_ is easily decomposed to form S and W elements during laser cladding due to its low decomposition temperature, in which S can react with Cr to form CrS in the molten pool . In addition, Stellite 6 is a common Co-based alloy powder with a high Cr content, which is favorable for the reaction to form CrS. Therefore, we select Stellite 6 as metal matrix to synthesize CrS with WS_2_. As a common hard alloy additive, NbC possesses high melting point (~3600 °C), high hardness (~19.6 GPa), and a density of 7.79 g/cm^3^ (which is close to Co, ~8.9 g/cm^3^) [21]; because of these advantages, NbC was selected as additive with the purpose of improving CrS formation and refining the microstructure of the metal matrix. In the present research, Co-based alloy powder (Stellite 6, powder size: about 75 μm), WS_2_ (particle size: 0.5–1 μm), and NbC (particle size: 1–3 μm) were used as cladding materials. The chemical composition of substrate and Stellite 6 were listed in Table 1. In order to better explore the microstructure evolution of the coatings, the cladding materials were made as mixtures of Stellite 6 with different contents of WS_2_ and NbC. The mixtures’ composition for coatings was illustrated in Table 2. The mixtures were dried at 80 °C for 30 min prior to being ground in a mechanical ball mill for 2 h to ensure a uniform dispersion.

### 2.2. Laser Cladding

**Figure 1:**
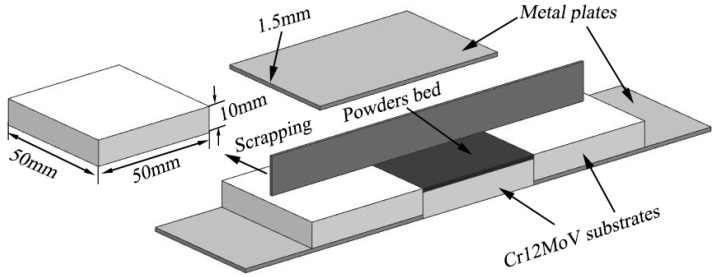
Schematic of preparation powders on substrate.

**Figure 2:**
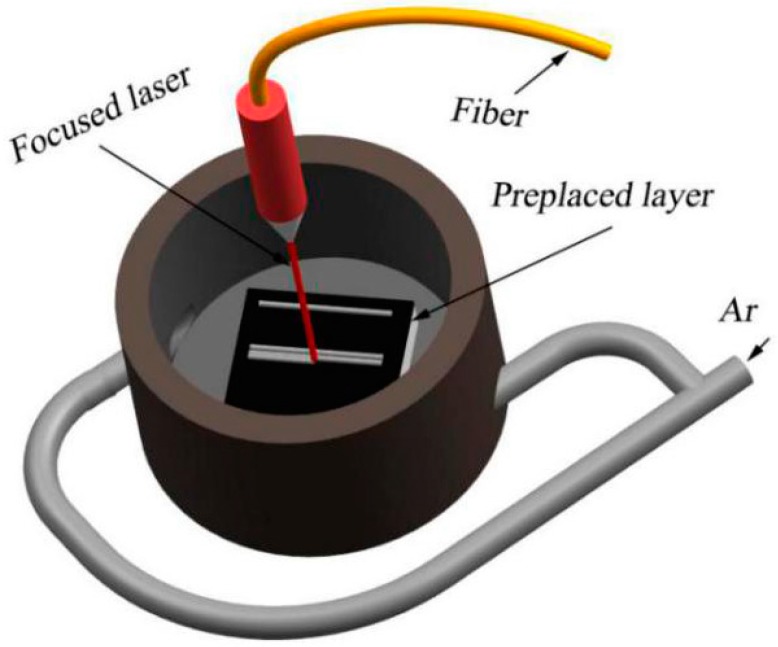
Schematic of laser cladding process.

Prior to laser cladding, the mixed powder needs to be prepared on the substrate. The bonding method is very simple and flexible, and commonly used to prepare the preplaced layer [22]. However, the binder used in the method may be decomposed to other elements and pollute the coating during laser cladding. In order to solve the shortcoming mentioned above, mixed powder was preplaced on substrate without using the binder. The preplace process is illustrated in Figure 1 and the thickness of preplaced layer was controlled by about 1.5 mm. Laser cladding was carried out using a IPG-YLS-5000 fiber laser system in a novel gas protection device, and the laser cladding process is illustrated in Figure 2. The optimized parameters utilized in the experiments are as follows: laser power 2.5 KW, scanning speed 12.5 mm·s^−1^, and spot diameter 3 mm. Argon was selected as the protective gas with a flow rate 10 L·min^−1^. The protective gas was switched on three seconds before laser cladding procedure until three seconds after the procedure finished. Then the single track and overlapped coatings with an overlap rate of 40% were cooled in air.

### 2.3. Microstructure Characterization

The cross section specimens of the coated samples were prepared perpendicular to the single track with a wire cutting machine, then processed by a standard metallographic procedure, and finally eroded in a mixed solution (volume ration of HNO_3_:HCl: CH_3_COOH:H2O = 1:4:1:1) for 90 s. The microstructure and element distribution was characterized by scanning electron micro-scope (SEM, Hitachi/S-3400, Tokyo, Japan) equipped with an energy dispersive spectrometer (EDS). The crystal phases of coatings were identified by X-ray diffraction (XRD, PANalytical/X’Pert Pro, EA Almelo, The Netherlands). Surface morphology of the wear scars was characterized by scanning electron microscope (SEM). 

### 2.4. Microhardness and Wear Test

**Figure 3:**
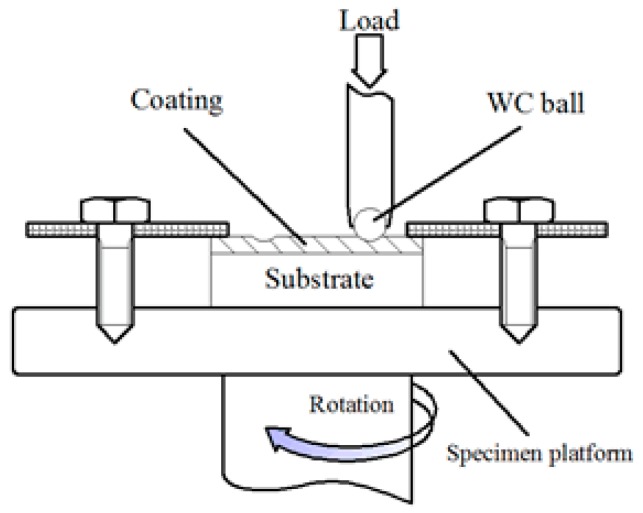
Schematic of wear test.

The microhardness along the depth direction of coating was measured by a Vickers microhardness tester (HXD-1000TMSC/LCD, Guangzhou, China) with a load of 500 gf and a dwell time of 15s; the microhardness was measured repeatedly and average was adopted. The dry sliding friction test was carried out on a pin-on-disc friction and wear testing machine (Bruker/UMT-3, Baden-Württemberg, Germany) in air for 30 min. The schematic diagram of friction and wear was illustrated in Figure 3. The counter-body was a WC ball with a diameter of 9.5 mm and a hardness of 1700 HV. The applied load was 10 kg and the sliding radius was 3 mm. The wear volumes of the coatings were measured using an optical microscopy (OM, KEYENCE/VHX-5000, Osaka, Japan). The wear rare *W* was calculated with the formula below:

(1) $$W=\frac{V}{LS}$$

where *V* is the wear volume (mm^3^), *L* is load (N) and *S* is the total sliding distance (m).

## 3. Results and Discussions

### 3.1. XRD Results

**Figure 4:**
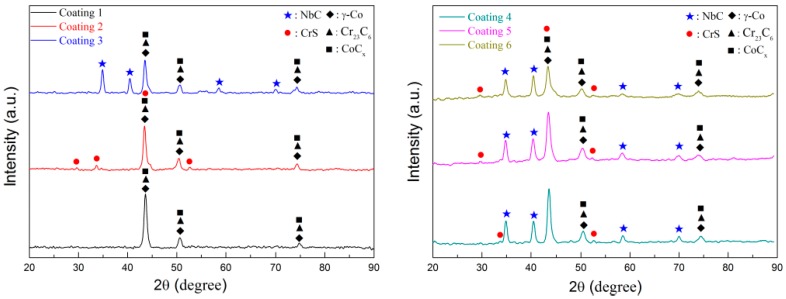
XRD (X-ray diffraction) patterns of the coatings.

The XRD patterns of the coatings are shown in Figure 4. It was found that coating 1 (Stellite 6) is mainly comprised of γ-Co, CoC_x_, and Cr_23_C_6_. When 15 wt % WS_2_ is added in Stellite 6, some new peaks located at 29.8°, 33.8°, and 53.4°are found in coating 2. They are conformed as the peaks of CrS in terms of the relevant JCPDS card (No. 03-065-9010 for CrS). When 30 wt % NbC is added in Stellite 6, some other new peaks located at 34.8°, 40.4°, 58.5°, and 69.9°are found in coating 3. They are conformed as the peaks of NbC in terms of the relevant JCPDS card (No. 03-067-7964 for NbC). Compared with the XRD data of CrS in Ref. [19] and NbC in Ref. [23], it can be deduced that coating 4, coating 5, and coating 6 mainly consists of γ-Co, CoC_x_, Cr_23_C_6_, CrS, and NbC. Comparing XRD patterns of coating 1, coating 3, and coating 5, it is indicated that NbC remain exist in the coating during laser cladding process. However, no WS_2_ phase can be found in XRD analysis. It indicates that WS_2_ decomposed into W and S elements during laser cladding process, in which S reacted with the melting Cr to form CrS in the molten pool [19]; thus, CrS appears in the coating 2, coating 4, coating 5, and coating 6.

### 3.2. Microstructure of the Coatings

**Figure 5:**
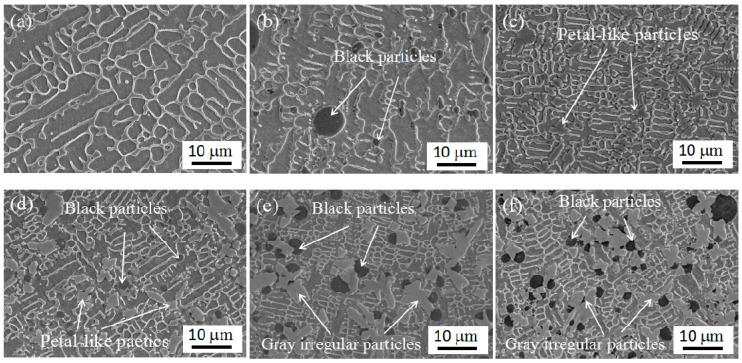
SEM (scanning electron microscopy) images of coatings: (**a**) coating 1, (**b**) coating 2, (**c**) coating 3, (**d**) coating 4, (**e**) coating 5, and (**f**) coating 6.

**Figure 6:**
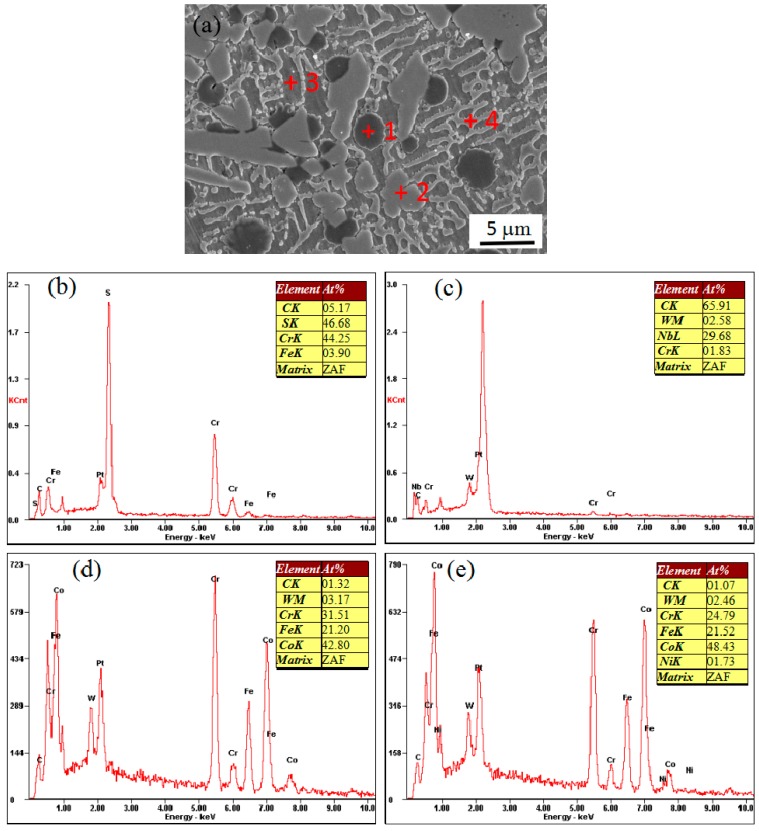
EDS (energy dispersive spectrometer) spot analysis: (**a**) typical microstructure, (**b**) Point 1, (**c**) Point 2, (**d**) Point 3, and (**e**) Point 4.

Typical SEM micrographs of the coatings are shown in Figure 5. It has been observed that the microstructure of the coating 1 was mainly composed of network dendritic structure and column -like crystals (Figure 5a). When 15 wt % WS_2_ was added in Stellite 6, some black particles with different size were found in coating 2, as shown in Figure 5b. When 30 wt % NbC was added to Stellite 6, petal-like particles from large to small were detected right next to each other, along with refined network dendritic structures that appear in coating 3, as shown in Figure 6c. By the same laser processing conditions, when 30 wt % NbC and different amounts of WS_2_ (such as 10%, 15%, and 20%, corresponding to coating 4, coating 5, and coating 6) were mixed with Stellite 6, Black particles, petal-like particles, gray irregular particles, and refined structure all appeared in those coatings. In addition, the size and the distribution of black particles are more uniform. Moreover, the amount of the black particles increased with the increase in addition of WS_2_. Based on the above results, it can be found that the addition of NbC mainly causes two changes in microstructure, corresponding to refining the microstructure of the metal matrix and improving size, distribution, and content of the black particles. When the amount of NbC added is fixed, with an increase in addition of WS_2_, the content of the black particles increases.

A SEM image at a higher magnification of coating 5 reveals that the coating is composed of four phases (as shown in Figure 6a), corresponding to black, nearly circular particles; gray irregular particles; network dendrites; and the matrix. EDS was used to identify the chemical compositions of these morphologically different phases. The elements’ composition is shown in Figure 6b–e. Based on the result of EDS analysis at point 1, it can be found that black nearly circular particles in coating 5 are enriched in Cr and S, and the ratio of Cr to S atoms is approximately 1:1, which was identified as CrS. Based on the results of point 2, gray irregular particles are enriched in Nb and C, which were identified as NbC. Based on the results of point 3 and point 4, the dendrite crystal and the phase between dendrites were mainly composed of Co, Cr, and Fe; it was identified as γ–Co and the solid solution of Co, Cr, and Fe.

**Figure 7:**
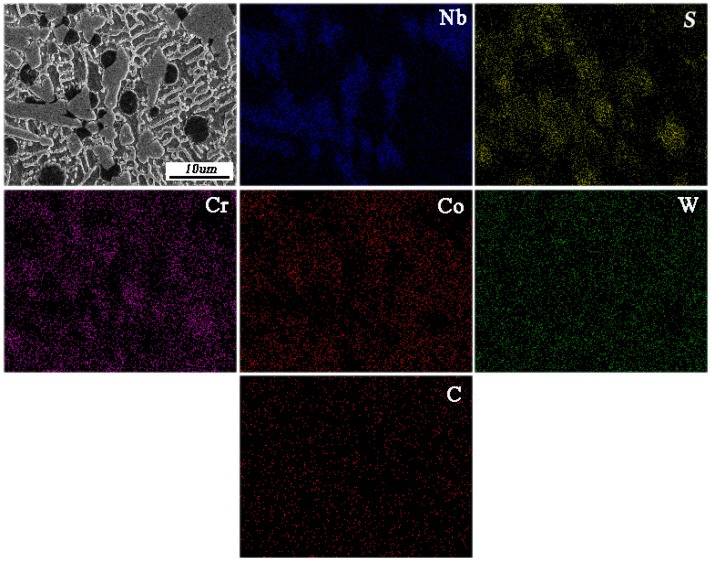
EDS elemental maps and atomic concentration of coating 5.

**Figure 8:**
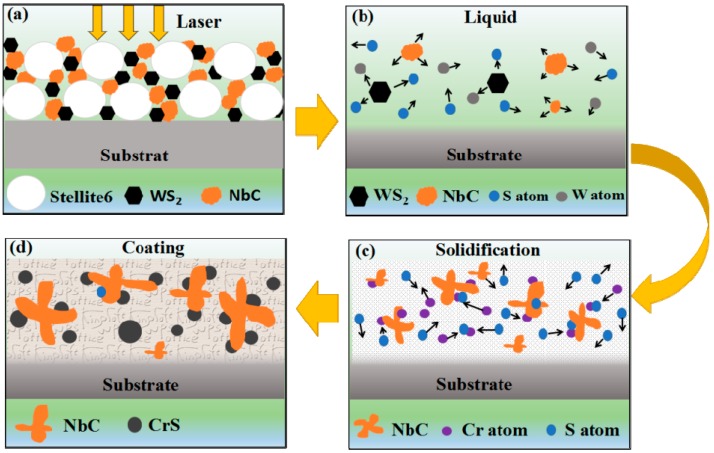
Schematic diagram of CrS and NbC evolution mechanism: (**a**) laser beam irradiates the surface of the preplaced layer; (**b**) WS_2_ decompose to S and W elements and NbC dissolve in the molten pool; (**c**) NbC precipitation form molten pool and CrS nucleation; (**d**) NbC and CrS distributed evenly in the coating.

To explore the formation mechanism of NbC and CrS, EDS mapping analysis was carried out to obtain element distribution. The results are shown in Figure 7. As can be seen, Nb element is distributed in the gray irregular particles, S element and Cr element are mainly distributed in the black nearly circular particles, Co element is distributed around the gray irregular particles and black nearly circular particles, W element is homogenously distributed, and the C element show no signs of enrichment. Combining the above XRD analysis in Figure 4 and microstructure analysis in Figure 6, the possible formation mechanism of CrS and NbC is presented in Figure 8. Firstly, laser beam irradiated the surface of the preplaced layer and most of laser energy was absorbed by mixed powder (as shown in Figure 8a). Then, the temperature of the coating increases rapidly, when the temperature reached the melting point of clad materials, the Stellite 6 powders were melted, WS_2_ was decomposed to S and W elements. As the temperature is elevated continuously, NbC was dissolved in the molten pool (as shown in Figure 8b). When laser beams scan out from the molten pool, the temperature decreases quickly; NbC was primarily the precipitation form of molten pool due to its higher melting point. As the temperature decreases continuously, CrS begins to nucleate spontaneously or attach to the surface of NbC nucleation (as shown in Figure 8c). Finally, NbC and CrS grow up in the process of solidification and are distributed evenly in the coating (as shown in Figure 8d) [24].

**Figure 9:**
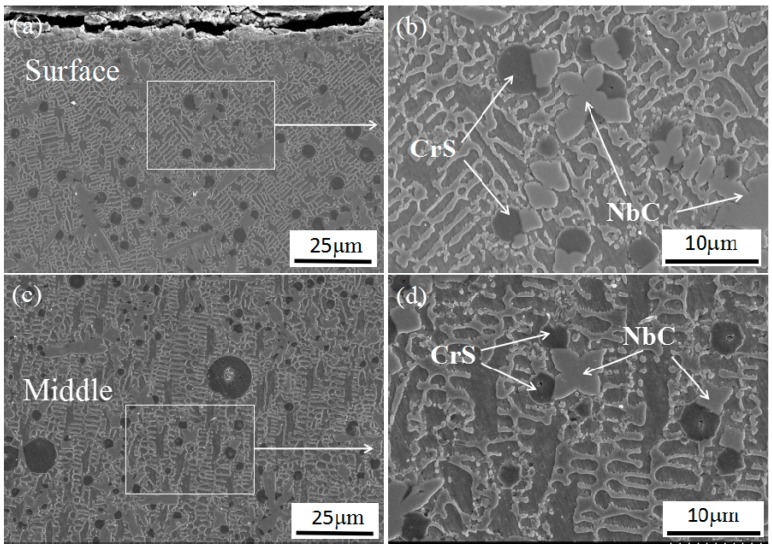

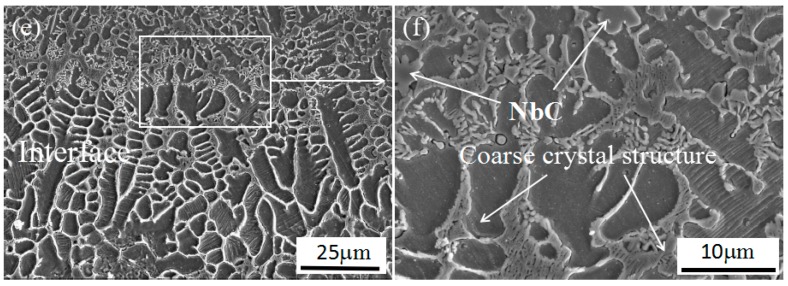
SEM images of cross-section of coating 5: (**a**,**b**) top region, (**c**,**d**) middle region, (**e**,**f**) bottom region.

To further characterize the coatings features, SEM micrographs of the coating 5 in different regions are provided in Figure 9. The microstructure in upper region is shown in Figure 9a. It can be found that NbC and CrS are distributed evenly in the coating. A larger SEM magnification is shown in Figure 9b for investigating the particles features. It is seen that most CrS attach to the surface of NbC, which indicates that CrS attach to the surface of NbC particles for nucleation and growth during the solidification process. The microstructure in middle region is shown in Figure 9c. It has a similar microstructure to the upper region. However, when comparing the size of CrS and other phases carefully, it was found that a few larger CrS particles appeared in the middle region (as shown in Figure 9c). As the temperature gradient decreases from the surface to the center of the molten pool, the CrS particles have more time for growth and hence a few CrS particles grow abnormally during solidification. The microstructure in the bottom region is shown in Figure 9e,f. Bright white, coarse crystal structure appears in the interface due to the bottom region was diluted by substrate. Besides, no CrS particles are distributed in the bottom region; only a few smaller NbC particles are located near the interface.

### 3.3. Microhardness

**Figure 10:**
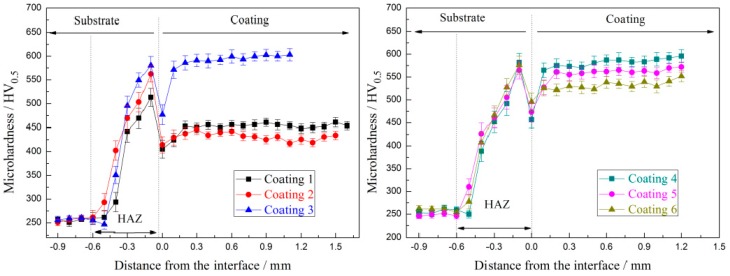
Microhardness distribution across the cross-section of the coatings.

Microhardness profiles across the cross-section of the coatings are shown in Figure 10. It can be clearly seen that the coating with 30 wt % NbC added in Stellite 6 has the highest microhardness (598.2 HV_0.5_ for coating 3), which is attributed to the high-hardness reinforcement of NbC uniform distribution in the matrix [19]. Besides, the addition of NbC-induced fine microstructure is also favorable for the further improvement in hardness/strength of the coating. However, for the coating with 15 wt % WS_2_ added in Stellite 6, there is a slight decrease of the microhardness (431.2. HV_0.5_ for coating 2) compared with Stellite 6 coating (454.2 HV_0.5_ for coating 1). Moreover, fixed the content of NbC with 30 wt % and increasing gradually the content of WS_2_ from 10 wt % to 20 wt %, the average microhardness of the composite coatings present a decreasing tendency as analyzed above (587.3 HV_0.5_ for coating 4, 546.6 HV_0.5_ for coating 5, and 534.6 HV_0.5_ for coating 6). According to the mixture principle of composites, the addition of soft material in hard matrix can decrease the hardness of a metal-matrix composite material [25]. CrS is relatively soft compared with metal matrix. It was noticed that the microhardness of the coating near the interface dramatically decreased; this phenomenon is similar to Ya’s report [26]; coarse structure and little reinforced NbC phase (as shown in Figure 10) are the main reasons that lead to hardness decrease. It was also found that the microhardness of all coatings increases sharply in the heat affected zone (HAZ), which should be attributed to the fact that substrate was quenched during laser cladding process due to the high cooling rate [23].

### 3.4. Tribological Behavior

**Figure 11:**
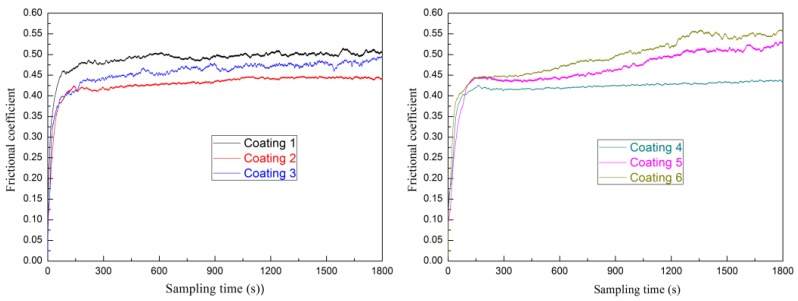
The changes in friction coefficient with the sliding time of coatings.

Figure 11 shows the changes in friction coefficient of the coatings against a WC ball under dry sliding condition at ambient temperature. In contrast, the frication coefficient of coating 1 and coating 3 fluctuate greatly with the sliding time. The frication coefficient of coating 2 and coating 4 vary a little with the sliding time. Moreover, their friction coefficient was stable during in dry sliding wear testing. For coating 5 and coating 6, their friction coefficient presents an increasing trend with sliding time and fluctuates greatly at the later period of sliding friction test. This may be caused by the wear device (see Figure 3). Due to the limited of friction device, the debris is not easy to discharge from the friction zone; part of the falling debris crushed into the friction area, resulting in increased fluctuation of the friction coefficient.

**Figure 12:**
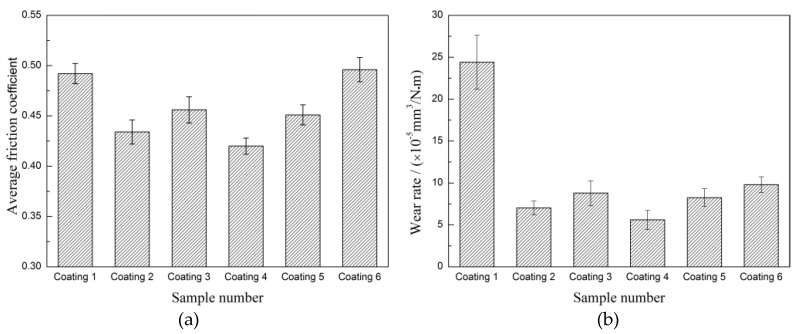
Friction coefficient (**a**) and wear rates (**b**) of coatings.

**Figure 13:**
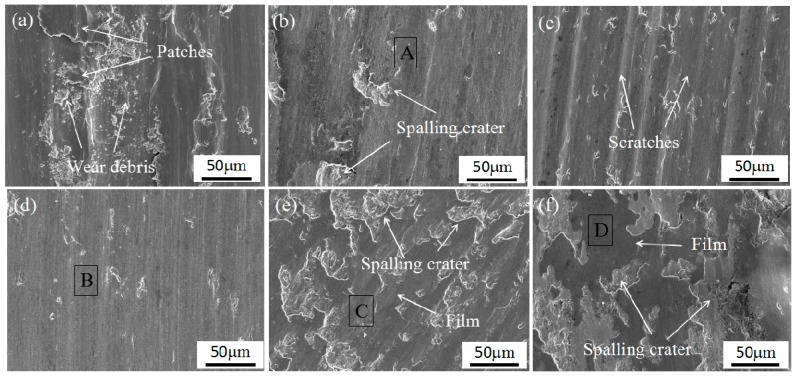
SEM micrographs of the worn surfaces of coatings: (**a**) coating 1, (**b**) coating 2, (**c**) coating 3, (**d**) coating 4, (**e**) coating 5, and (**f**) coating 6.

The average friction coefficient and wear rate of the coatings were also calculated (seeing Figure 12). The friction coefficient of coating 2 (0.434) and coating 3 (0.456) are lower than the coating 1 (0.489); moreover, the wear rate of coating 2 (7.02 × 10^−5^ mm^3^/N·m) and coating 3 (7.86 × 10^−5^ mm^3^/N·m) are far less than coating 1 (2.43 × 10^−4^ mm^3^/N·m). It indicated that both CrS and NbC contribute to improving the tribological properties of the coatings. The wear performance of the coatings is closely related to the microstructural evolution resulting from the addition of WS_2_ and NbC as discussed in Section 3.2. The synthetic self-lubrication phase CrS is favorable for the composite coating’s anti-wear and friction reduction capabilities [14,19]. Moreover, the addition of NbC can not only lead to microstructural fineness, but also result in a large number of ceramic particles distribute in the coating, which endow the coating with excellent wear resistance [21,27]. However, there are no similar reports about the comprehensive impact of CrS and NbC on the wear behavior of laser clad coatings. Although the coating 4, coating 5, and coating 6 also exhibited good wear resistance as shown in Figure 12, the average friction coefficient of coating 4 (0.421), coating 5 (0.451), and coating 6 (0.495) shows an upward trend with the increase in the addition of WS_2_. To further characterize the CrS and NbC on the tribological behavior of these types of coatings, SEM micrographs of the worn surfaces are provided in Figure 13. There were some wear debris, patches, and serious plastic deformation on worn surface of coating 1 (as shown in Figure 13a); this illustrated that the coating suffered severe adhesive wear. The worn surface of coating 2 is very smooth except for some spalling craters (as shown in Figure 13b). According to the EDS result of position A (listed in Table 3), the worn surface of the coating 2 composed of Cr, Co, Fe, and S indicated CrS has been released and smeared on the wear surface due to its inherent soft and solid lubricant character, which can reduce the friction coefficients between the friction pair interface [14,28]. The worn surface of coating 3 is relatively smooth with only slight scratches characteristics (as shown in Figure 13c), which indicates that the slight abrasive wear occurred. This should be attributed to the high hardness and refined microstructural of the coating that make it very difficult to be plastically deformed or plowed during the dry sliding wear [29,30]. The worn surface of coating 4 is very smooth and has no spalling crater, plastically deformation, or scratches on it (as shown in Figure 13d). It shows that coating 4 has good tribological properties. However, the worn surface of coating 5 and coating 6 are obviously different to coating 4; black traces of lubricious film and larger spalling crater are visible on the worn surface (as shown in Figure 13e,f). According to the EDS result of position B, C, and D (listed in Table 3), the worn surface of coating 4, coating 5, and coating 6 composed of Cr, Co, Nb, and S elements indicated that hard NbC and lubricant CrS have been released on the worn surface. Though the content of CrS is higher than the increase in the addition of WS_2_, but more CrS and larger NbC were crushed into the friction area, resulting in a lot of spalling craters on the wear surface. Worse still, these spalling craters were harmful to forming continuous transfer film, resulting in an increase and a fluctuation of the friction coefficient [15,31]. In fact, tribological performance is a comprehensive property in relation to both the lubrication and the other mechanical parameters of materials. Additionally, the suitable balance of CrS and NbC is favorable to decreasing friction and improving stability of the contact surfaces between the WC ball and the coatings. In this research, coating 4 shows the best tribological properties.

## 4. Conclusions

(1) Co-based metal matrix self-lubricating coatings containing solid-lubricant phase CrS and ceramic reinforced phase NbC were successfully fabricated on Cr12MoV steel surface by laser cladding Stellite 6, WS_2_, and NbC matrix powder.
(2) Reactions between WS_2_ and Stellite 6 occurred, which generated solid-lubricant particles CrS and NbC that play a key role in improving the CrS nuclear and refining microstructure of the Co-based composite coating during laser cladding processing.
(3) The average microhardnesses of coating 4, coating 5, and coating 6 are 587.3 HV_0.5_, 546.6 HV_0.5_, and 534.6 HV_0.5_, respectively. NbC reinforcements and fine microstructure contribute a higher level of microhardness. The formation of soft CrS and the coarse NbC particles result in a slight decrease in microhardness.
(4) Both CrS and NbC contribute to improving the tribological properties of the coating, and the suitable balance of CrS and NbC favorable to decreasing the friction and improving the stability of the contact surfaces between the frictional pair and the coatings. In this research, coating 4 shows the best tribological properties.

## Figures and Tables

**Table 1:** Chemical composition of substrate and Stellite 6.

Material	Element (wt %)									
	C	Cr	Si	W	Mo	Ni	Mn	V	Co	Fe
Substrate	1.61	12.02	0.23	-	0.52	0.09	0.14	0.22	-	Bal.
Stellite 6	1.15	29.00	1.10	4.00	1.00	3.00	0.05	-	Bal.	3.00

**Table 2:** Mixtures composition for coatings.

Number	Mixtures Composition (wt %)		
	Stellite 6	WS_2_	NbC
**Coating 1**	100	-	-
**Coating 2**	85	15	-
**Coating 3**	70	-	30
**Coating 4**	60	10	30
**Coating 5**	55	15	30
**Coating 6**	50	20	30

**Table 3:** EDS-determined compositions of the regions on the worn surfaces marked in Figure 13.

Point No.	Element (at. %)						
	S	Cr	Nb	C	Fe	W	Co
A	9.37	27.01	-	1.40	22.76	2.93	36.53
B	7.39	16.40	19.77	5.95	15.74	3.22	31.73
C	10.05	21.73	15.93	3.09	17.32	3.04	28.82
D	13.68	25.59	9.23	2.57	18.74	3.39	26.53
